# Chromium(III) substitution inhibits the Fe(II)-accelerated transformation of schwertmannite

**DOI:** 10.1371/journal.pone.0208355

**Published:** 2018-12-05

**Authors:** Girish Choppala, Edward D. Burton

**Affiliations:** Southern Cross GeoScience, Southern Cross University, Lismore, New South Wales, Australia; The University of Akron, UNITED STATES

## Abstract

Schwertmannite is an Fe(III)-oxyhydroxysulfate which is common in acid mine drainage (AMD) and acid sulfate soil (ASS) environments. Natural schwertmannite is often enriched in Cr(III), yet the effects of Cr(III) substitution on schwertmannite transformation to more stable Fe(III) minerals has not been addressed. Here we examine, for the first time, the effects of Cr(III) substitution on the Fe(II)-accelerated transformation of schwertmannite. X-ray diffraction (XRD) and Fe K-edge extended X-ray absorption fine structure (EXAFS) spectroscopy shows that Cr(III) substitution inhibits schwertmannite transformation. Substitution at a Cr(III):Fe(III) ratio of 0.025 decreased schwertmannite transformation (at pH 6.5) by 18–49% (depending on Fe(II) concentrations) relative to that of Cr(III)-free schwertmannite. Formation of crystalline secondary phases (predominantly goethite) caused associated decreases in solid-phase Fe and Cr extractability by 1 M HCl. The extractability of Cr was consistently greater than that of Fe, suggesting some accumulation of Cr(III) at the residual schwertmannite surface–a phenomenon which passivates the surface against Fe(II)/Fe(III) electron transfer and atom exchange required for the Fe(II)-accelerated transformation process. The finding that Cr(III)-substitution inhibits schwertmannite transformation implies that it may also significantly impact associated Fe, S and trace metal(loid) behaviour.

## Introduction

Schwertmannite is a poorly-ordered Fe(III)-oxyhydroxysulfate which precipitates from acidic, Fe- and SO_4_^2—^rich waters [1–3]. As such, it occurs commonly in systems that are affected by acid-sulfate soils (ASS) and acid-mine drainage (AMD) [4–9]. The formation and fate of schwertmannite strongly influences Fe and S cycling, the generation and consumption of acidity, as well as the mobility of trace metals and metalloids [4, 5, 10–13].

Schwertmannite is metastable, transforming over time to more stable Fe(III) oxides, such as goethite (α-FeOOH) [9]. Although metastable, schwertmannite tends to persist under acidic, oxic conditions for periods of time ranging from several months to years [14]. However, it’s transformation is greatly accelerated under near-neutral, anoxic conditions by interactions with Fe(II) [15–18]. The conditions required for this Fe(II)-accelerated transformation process can rapidly develop due to microbial Fe(III)-reduction when, for example, previously drained ASS wetlands are re-flooded [13, 16, 19–22]. Such conditions are also well documented for a range of AMD settings, including mine-pit lake sediments and organic-rich constructed wetlands [10, 23–26].

Natural precipitates of schwertmannite are often enriched in Cr (up to approx. 1000 mg kg^-1^) [27–30]. In aqueous environments, Cr predominantly exists as Cr(VI) and Cr(III) species [31]. Chromium(VI) is highly toxic and carcinogenic, and its interactions with schwertmannite have been relatively well-studied [29, 32, 33]. However, Cr(VI) is typically absent from acid-sulfate waters due to the rapid reduction of Cr(VI) to Cr(III) in the presence of elevated Fe(II) concentrations and low pH (which characterise such waters) [28]. As a consequence, schwertmannite which precipitates from Fe(II)-bearing, acid-sulfate waters is likely to contain Cr predominantly in the Cr(III) oxidation state.

The substitution of Cr(III) for Fe(III) in Fe(III) oxides is well-known, and its effects have been studied for many Fe(III) oxides including hematite, goethite and ferrihydrite [34–39]. However, Cr(III) substitution in schwertmannite has received little attention. In particular, no previous studies have addressed the potential effects of Cr(III) substitution on schwertmannite stability with regard to its Fe(II)-accelerated transformation. The associated effects on Cr mobility and fate during this transformation process have also not been examined. Given the ubiquity of Cr(III)-enrichment in natural schwertmannite, these potential effects of Cr(III) substitution represent an area requiring further research.

In this work, we examine the effects of Cr(III) substitution on the Fe(II)-accelerated transformation of schwertmannite. The results also provide insights into how this transformation process affects the associated availability of solid-phase Cr(III).

## Materials and methods

### General

All reagents were of analytical grade and all solutions were prepared using deoxygenated Milli-Q water (18 MΩ resistivity, Millipore Corp, Milford, MA, USA).

### Schwertmannite synthesis

Chromium(III)-free schwertmannite was synthesised following Burton et al. [40]. This involved dissolving 1500 g of FeSO_4_.7H_2_O (Sigma Aldrich, Australia) in 50 L of deionised water. To this solution, 800 mL of 30% H_2_O_2_ was added to initiate schwertmannite formation. The resulting suspension was allowed to settle for 24 h, followed by five rinsing cycles (with each cycle involving the replacement of about 80% of the supernatant solution with deionised water). The final schwertmannite slurry was dried at 40°C and finely ground.

Different levels of Cr(III) substitution in schwertmannite were achieved by following the same procedure as described above, except for the addition of differing quantities of Cr(VI) (K_2_CrO_4_, Sigma Aldrich, Australia). It should be noted that, under the synthesis conditions, all Cr(VI) is rapidly reduced to Cr(III), thereby leading to Cr(III) substitution into the resulting schwertmannite. Three contrasting levels of Cr(III) substitution (hereby termed low, medium and high) were prepared by the addition of 5.24, 13.1 and 52.4 g K_2_CrO_4_ respectively, to the FeSO_4_.7H_2_O solution prior to the addition of H_2_O_2_. The Cr(III)-substituted schwertmannite was dried and ground as described above for the Cr-free schwertmannite.

### Schwertmannite transformation experiment

Throughout this experiment, O_2_-free conditions were achieved via the use of an anaerobic chamber containing an atmosphere of 97–98% N_2_ and 2–3% H_2_. A constant weight (0.4±0.001 g) of schwertmannite was added to 50 mL polypropylene vials and mixed with 40 mL of anoxic 0.1 M NaCl buffered to pH 6.5 with 0.05 M MES/MOPS. This suspension was allowed to equilibrate for 24 h, after which pH was readjusted to 6.5 by addition of a 5.76 M NaOH solution.

Three levels of Fe(II) were achieved by addition of appropriate volumes of an FeCl_2_ stock solution, yielding an initial concentration of either 0, 1 or 10 mM Fe(II). The suspensions were placed on an orbital shaking table (120 rpm) within the anaerobic chamber, with quadruplicate subsamples removed periodically (at 1h, 12h, 1d, 2d, 4d, 7d and 14 d). At each sampling time, the suspensions were centrifuged (1472 *g*), and the supernatant solution was filtered to <0.45 μm. Immediately after filtering, aliquots of the aqueous phase were processed for determination of Fe(II), Fe(total), Cr(VI) and Cr(total). The solid-phase was rinsed with 40 mL deoxygenated methanol (via centrifugation and decanting), allowed to dry, ground using a mortar and pestle, and stored in glass vials sealed with rubber septa for further analyses (with all steps conducted under O_2_-free conditions).

### Aqueous phase characterisation

Iron(II) and Fe(total) fractions in the aqueous phase were analysed using the 1, 10-phenanthroline method [41]. Aqueous Cr(total) was analysed by ICP-OES (Perkin Elmer 4300DV, USA). Aqueous Cr(VI) was determined using the 1, 5 diphenylcarbazide method [42]. Chromium(VI) concentrations were below the limit of detection (<0.01 mg L^-1^) throughout the experiment.

### Solid phase characterisation

The concentration of Fe, S and Cr in the initial schwertmannite was analysed by ICP-OES (Perkin Elmer 4300DV, USA), after aqua-regia digestion. Changes in the extractability of solid-phase Fe(III) and Cr(III) during transformation experiment were also quantified via extraction with 1 M HCl for 1 hour.

Mineralogy was determined by X-ray powder diffraction (XRD) (Bruker AXS GmbH, Germany) using CoKα radiation. Powdered and homogenised samples were placed on either a standard sample holder or a low background quartz sample holder. X-ray diffractograms were acquired at 2*θ* angles from 10-80^o^, with a step size of 0.02^o^ and 2 second counting time. The diffraction patterns were evaluated using EVA software package (DIFFRAC-plus evaluation package, Bruker AXS, Germany).

The solid-phase micromorphology of individual particles was examined using scanning electron microscopy with energy dispersive X-ray spectroscopy (SEM-EDX, Zeiss EVOLS-15, Germany). The association of Cr(III) with specific minerals was examined using a Jeol JEM-2200FS transmission electron microscope (TEM). Samples for TEM examination were suspended in ethanol and disaggregated by grinding using a mortar and pestle. A drop of suspension was placed onto a carbon-coated Cu grid with a lacy-carbon support, allowed to air dry and then immediately loaded into a Jeol double-tilting, analytical specimen holder. Observations of various individual mineral particles were undertaken at a voltage of 200 kV.

Crystal lattice fringes were examined to provide data on lattice plane spacing and morphological features. Fast Fourier transform (FFT) analyses was performed using *Digital Micrograph* (Gatan Inc. USA) to measure d-spacings from selected areas. Semi-quantitative determination of key elements concentration ratios was carried out using Energy Dispersive X-ray spectrometry (EDX) with attached Oxford Link ISIS system (X-MAX 80 mm^2^). The EDX analyses was performed at three to four locations for each sample and averages for the compositions were calculated. Elemental mapping was also performed using TEM-EDX to observe the distribution of Cr(III) in the mineralogical transformation products.

Chromium and Fe K-edge X-ray absorption spectroscopy was conducted at Beamline 17C at the National Synchrotron Radiation Research Centre (NSRRC) in Hsinchu, Taiwan. Selected samples and reference phases were loaded in sample holders and sealed with Kapton tape. The XAS spectra of samples and standards were collected in fluorescence detection mode using a Si(111) double crystal monochromator. The storage ring was operating at 1.5 GeV in continuous injection mode. Data were averaged and normalised, with linear combination fitting (LCF) of the Fe K-edge EXAFS spectra performed over the k = 3–10 Å^-1^ range using the Athena software [43]. The combinatorics function in Athena was used to determine the best fit for all possible combinations from a library of Fe reference standards (prepared as described in Burton et al. [44]). Only components which contributed greater than 5% to the total were included in the final fits. This approach resulted in no more than 2–3 phases being required to fit any individual Fe K-edge EXAFS spectrum.

## Results and discussion

### Initial schwertmannite properties

**Fig 1:**
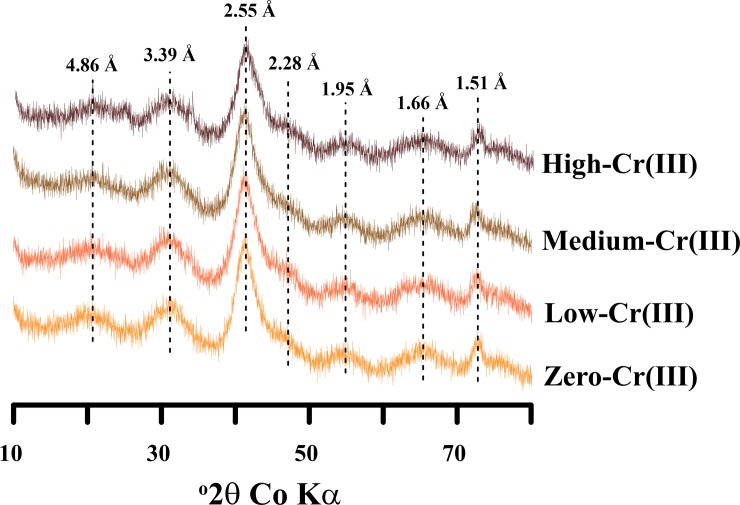
X-ray diffraction patterns of the initial schwertmannite specimens examined in this study. Schwertmannite peaks with *d*-spacing values are denoted with dotted lines.

The low-, medium- and high-Cr(III) schwertmannite displayed XRD patterns that were identical to Cr-free schwertmannite (Fig 1). The total Fe and S content of the initial schwertmannites ranged from 7.9–8.2 mmol g^-1^ and 1.63–1.82 mmol g^-1^, respectively (S1 Table), which is consistent with the compositional variability of natural schwertmannite [1, 4, 40, 45]. The total Cr content in the low-, medium- and high-Cr(III) schwertmannite was 0.02, 0.05 and 0.21 mmol g^-1^, respectively (S1 Table). These total Cr concentrations bracket those reported in previous studies on natural schwertmannite formed in AMD systems [27–30].

**Fig 2:**
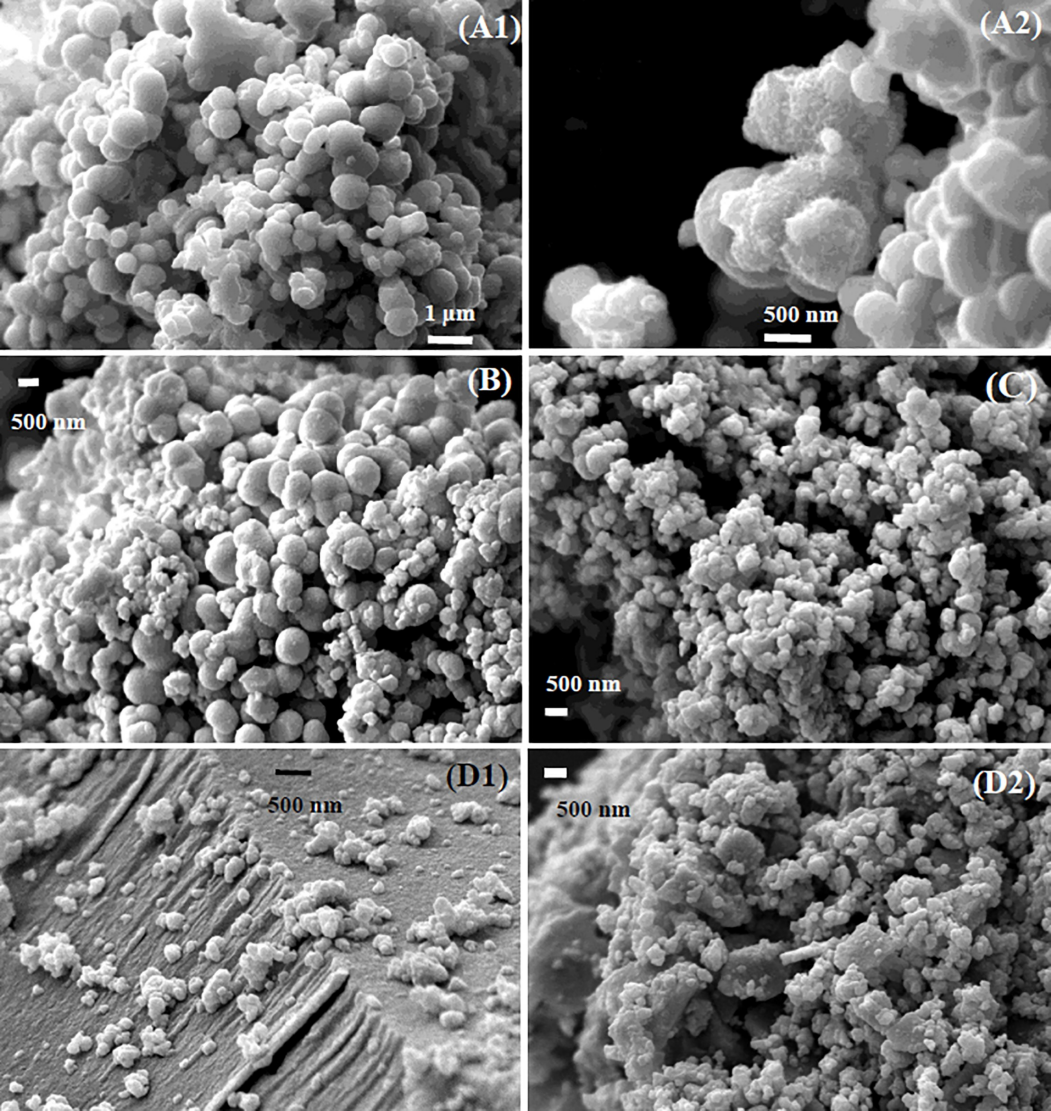
Scanning electron micrographs (SEM) of zero Cr(III)- and Cr(III)-incorporated schwertmannite. (A1, A2) zero Cr(III)- schwertmannite with large spheroids; (B) low; (C) medium and, (D1 and D2) high Cr(III)- incorporated schwertmannite.

Scanning electron microscopy revealed that the Cr-free schwertmannite existed as spheroids with a diameter of 400–600 nm (Fig 2). Increased levels of Cr(III) substitution was associated with decreases in the size of these schwertmannite spheroids (Fig 2A–2D) and with slight increases in the corresponding total SO_4_^2-^ content (S1 Table). This latter effect is consistent with the smaller schwertmannite spheroids having greater surface area, thereby enabling larger levels of SO_4_^2-^ adsorption.

**Fig 3:**
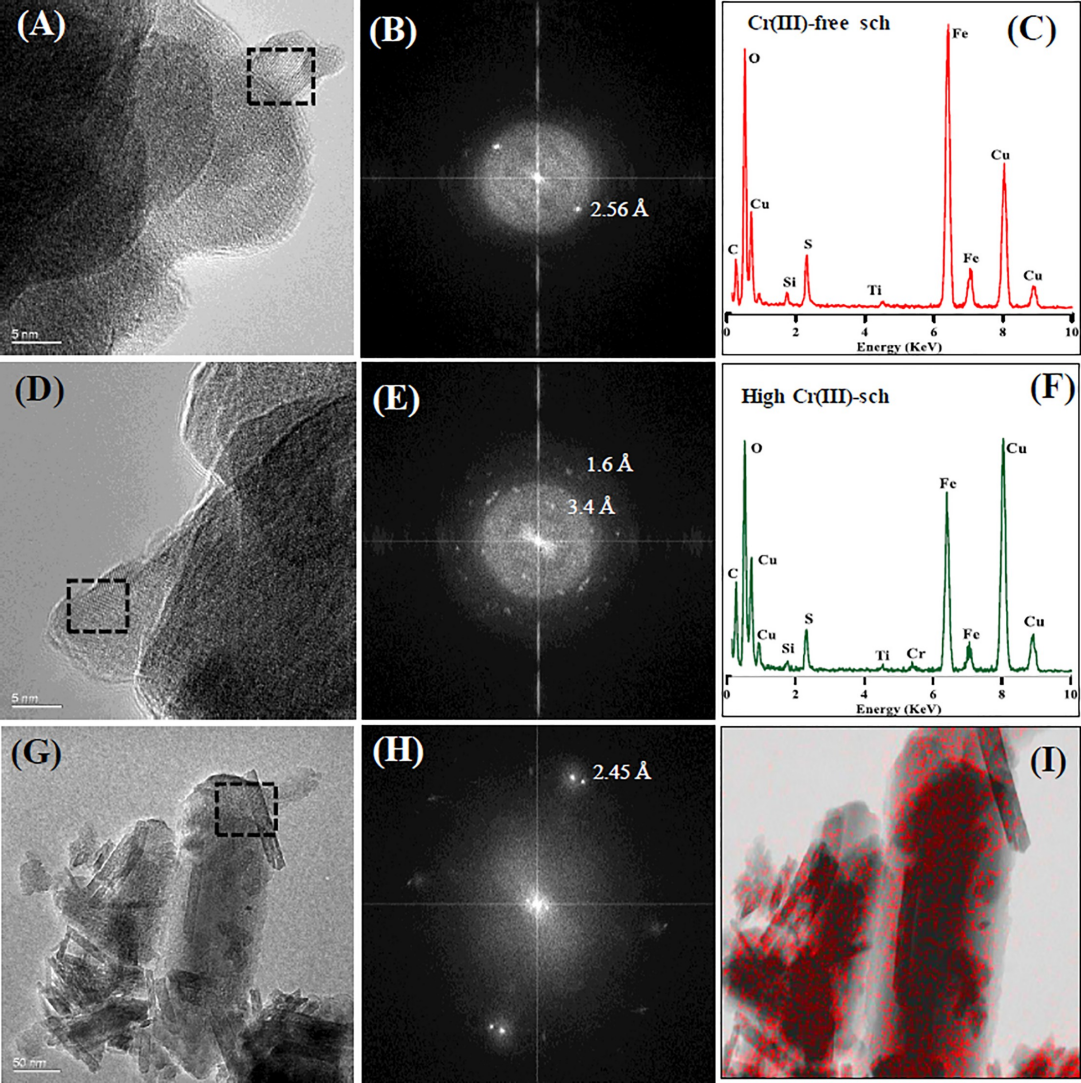
HRTEM micrographs. (A) Cr(III)-free schwertmannite; (B) FFT pattern of area enclosed by dashed-line in (A); (C) TEM-EDX spectrum of zero Cr(III)-schwertmannite; (D) high Cr(III)-schwertmannite; (E) FFT pattern of the area enclosed by dashed-line in (D); (F) EDX spectrum of high Cr(III)-schwertmannite; (G) high Cr(III)-schwertmannite sample that was treated with 10 mM Fe(II) after 14 days, (H) corresponding FFT pattern of the area enclosed by dashed line in (G) and (I) elemental mapping of sample shows Cr distribution.

High-resolution TEM indicated that the initial Cr(III)-free schwertmannite contained nano-crystalline domains, which were slightly elongated with visible lattice fringes (Fig 3A). Fast Fourier Transform (FFT) patterns of these nano-domains revealed d-spacing of 2.25 and 2.56 Å, consistent with schwertmannite (Fig 3B). TEM-EDX analyses revealed that Cr(III) substitution increased the abundance of nano-crystalline domains (Fig 3C and 3D), which was confirmed by FFT analyses (Fig 3E). EDX analyses of the initial high-Cr(III) schwertmannite show that Fe, O, S and Cr were homogenously distributed at the sub-micron scale (Fig 3F to 3I).

**Fig 4:**
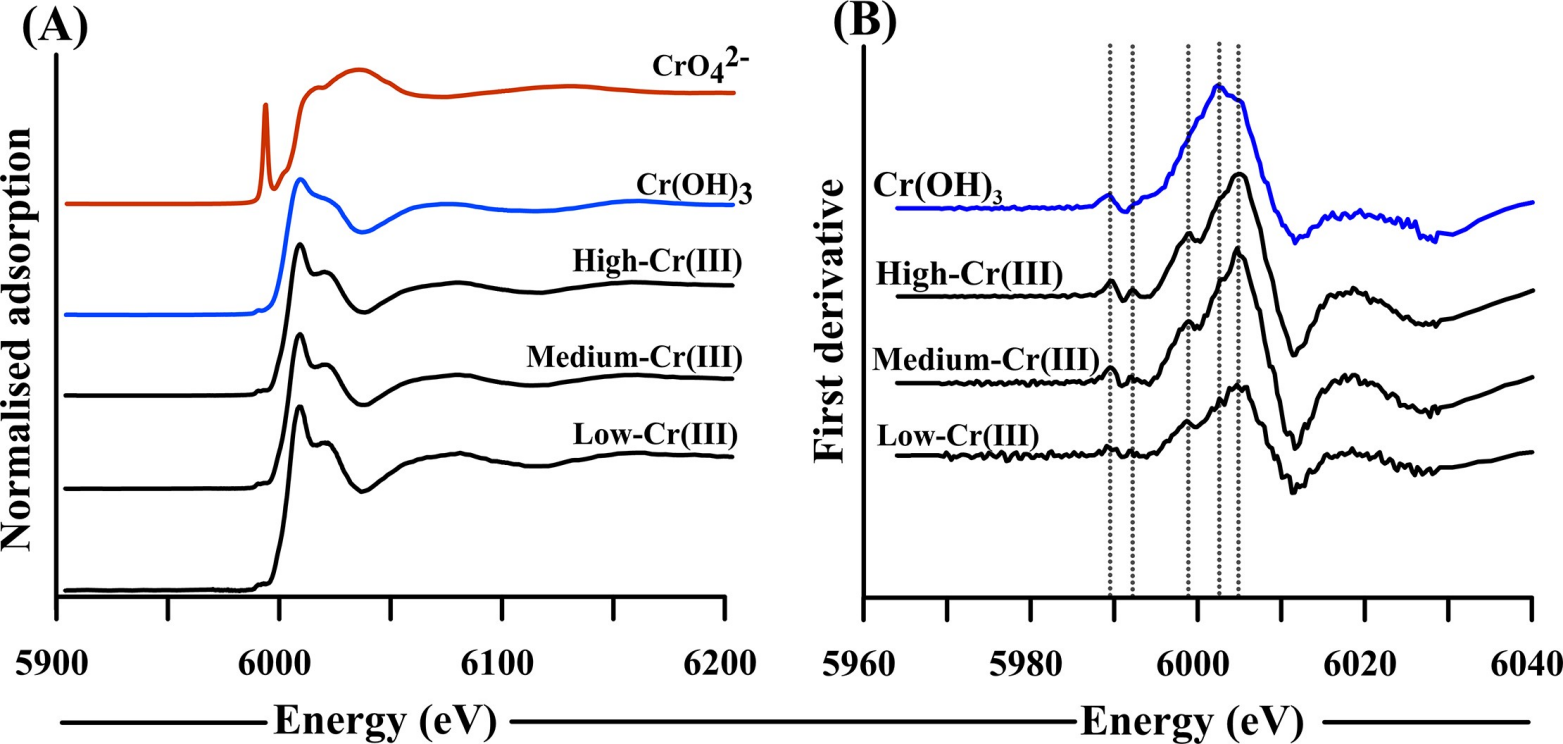
**Chromium K-edge XANES spectra of (A) Cr(III)-substituted schwertmannite, Cr(OH)**_**3**_ **and CrO**_**4**_^**2-**^ **and (B) corresponding first derivative spectra.** The vertical dotted lines in (B) highlight features which are attributable to Cr(III) for Fe(III) substitution based on Tang et al. [37].

Cr K-edge XANES spectroscopy showed that the Cr(III)-substituted schwertmannite contained Cr(III) with no Cr(VI) (Fig 4A). This is evident from the absence of a pre-edge peak at ~5989 eV in the Cr K-edge XANES spectra, which (when present) is indicative of Cr(VI). Accordingly, the XANES edge position of the Cr(III)-substituted schwertmannite was consistent with that of the Cr(OH)_3_ reference material. However, the first derivative of the XANES spectra revealed the presence of additional edge features for the Cr(III)-substituted schwertmannite (Fig 4B). These additional features were also observed by Tang et al. [37] for Cr(III)-substituted ferrihydrite, who attributed such features to Cr(III) substitution for Fe(III). There Cr K-edge XANES features provide evidence of Cr(III) substitution into the schwertmannite crystal structure (as opposed to the presence of discrete Cr(OH)_3_ crystallites mixed with Cr(III)-free schwertmannite crystallites).

### Schwertmannite stability in the absence of Fe(II)

**Fig 5:**
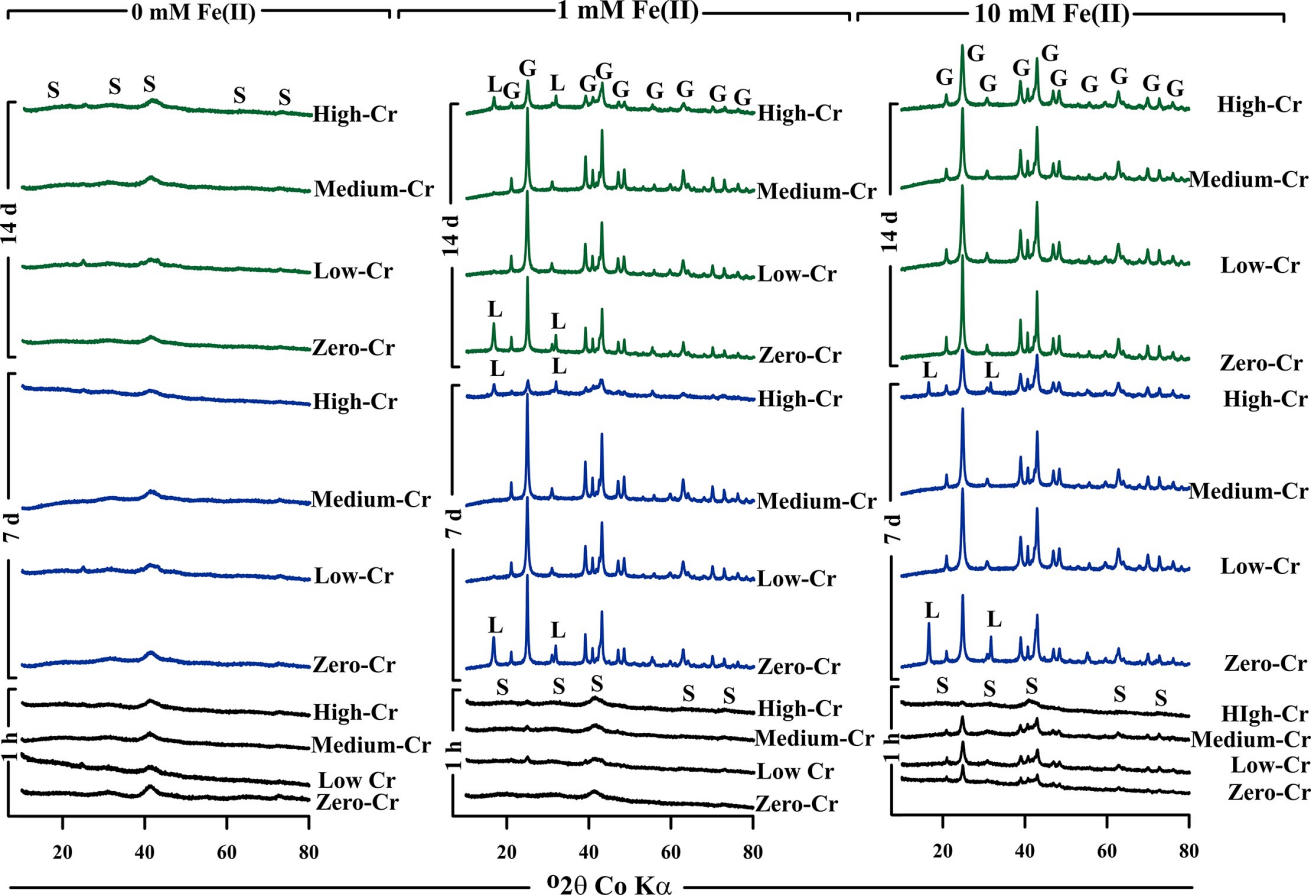
XRD patterns of material collected after allowing the zero-, low-, medium- and high-Cr(III) schwertmannite to react with 0, 1 or 10 mM Fe(II) over 1 h, 7 d and 14 d. Peaks for schwertmannite, goethite and lepidocrocite are denoted as S, G and L, respectively.

**Fig 6:**
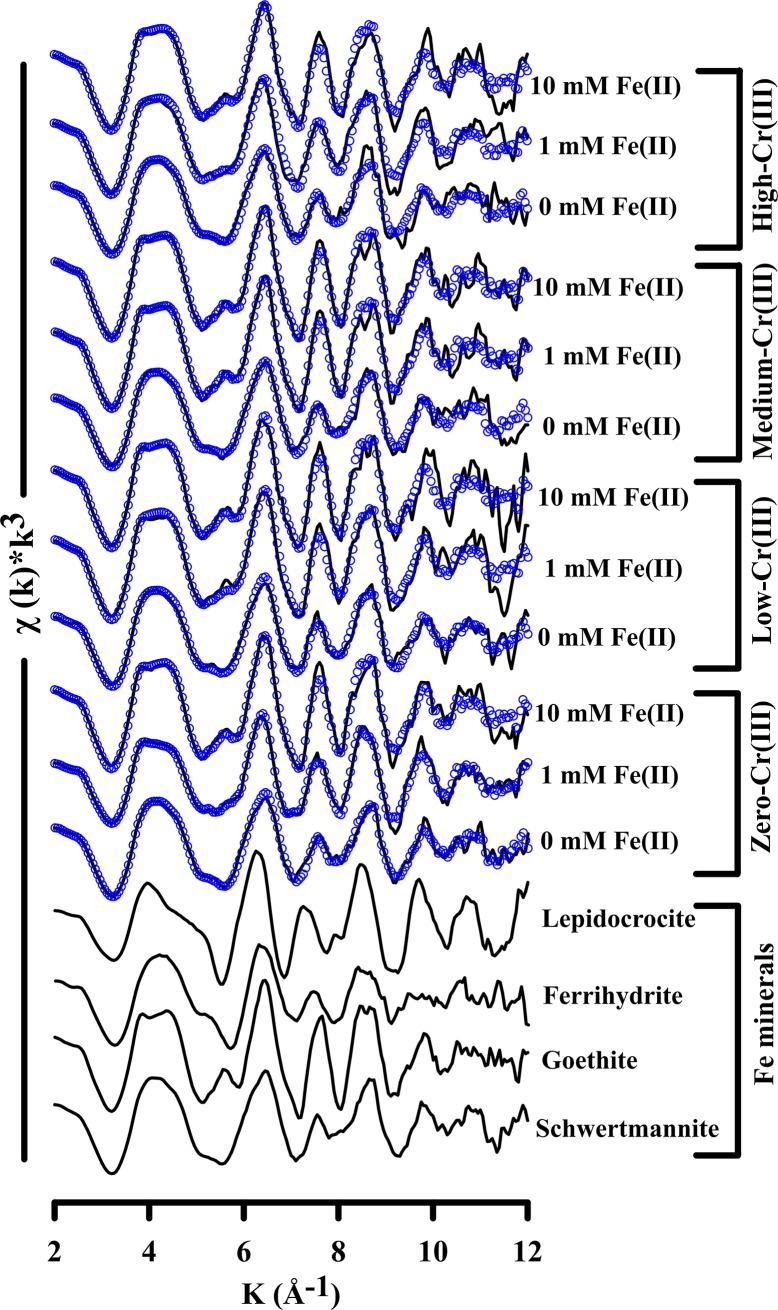
Iron K-edge EXAFS spectra of Cr(III)-free and Cr(III)-incorporated schwertmannite and their mineralogical transformation during Fe(II) accelerated transformation at reaction period of 14 days. The XAS data of the samples are shown as a solid line and the linear combination fit is shown as coloured circles.

**Fig 7:**
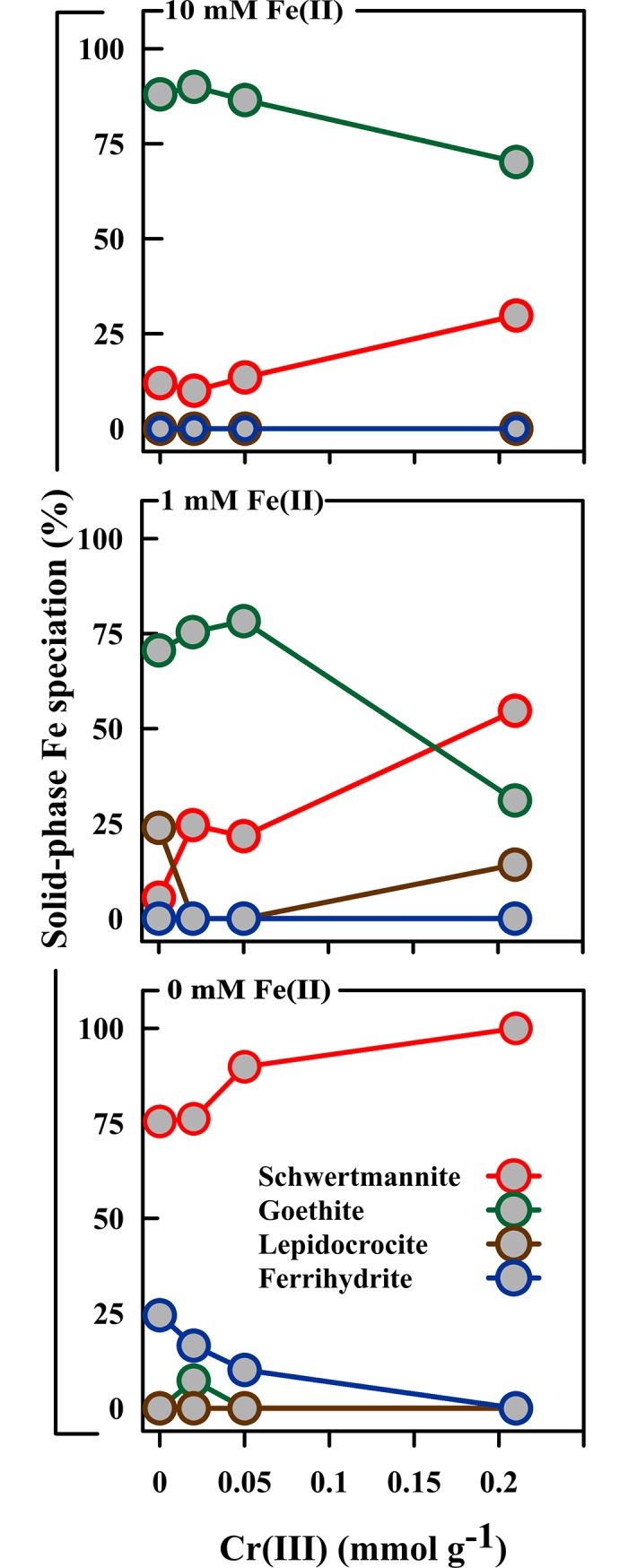
Solid-phase Fe speciation (based on Fe K-edge EXAFS spectroscopy) after allowing schwertmannite, with varying levels of Cr(III)-substitution, to react with 0, 1 and 10 mM Fe(II) for 14 d.

The XRD patterns show that, in the absence of Fe(II), schwertmannite experienced very little or no transformation to crystalline phases over the 14 day experiment duration (Fig 5). However, linear combination fitting of the corresponding Fe K-edge EXAFS spectra revealed some transformation of schwertmannite to ferrihydrite under Fe(II)-free conditions (Figs 6 and 7). The formation of ferrihydrite is consistent with previous work by Davidson et al. [46] who showed that ferrihydrite can form as an intermediate phase following the release of SO_4_^2-^ from schwertmannite.

The extent of ferrihydrite formation under Fe(II)-free conditions decreased with increases in the level of Cr(III) substitution in the initial schwertmannite (Fig 7). In this case, 24% of solid-phase Fe in the Cr(III)-free schwertmannite had transformed to ferrihydrite by day 14. In comparison, 16, 10 and 0% transformation occurred over 14 days for the low-, medium- and high-Cr(III) schwertmannite, respectively. The indistinguishable formation, based on XRD, of these amounts of ferrihydrite is consistent with ferrihydrite occurring as poorly-ordered nanocrystallites which produce only very diffuse XRD peaks which overlap those of schwertmannite.

### The Fe(II)-accelerated transformation of schwertmannite

The XRD patterns show that Fe(II) accelerated the transformation of schwertmannite to goethite, along with minor lepidocrocite in some samples (Fig 5). These patterns show that, as expected, the extent of secondary Fe(III) (oxy)hydroxide formation increased over time with increases in the Fe(II) concentration. This is consistent with previous studies showing that Fe(II) accelerates the transformation of schwertmannite to goethite and lepidocrocite [15–18, 47, 48]. The formation of lepidocrocite in some samples following the addition of Fe(II), and its subsequent disappearance by day 14 in the 10 mM Fe(II) treatment is consistent with lepidocrocite being an intermediate phase, which eventually transforms to goethite in the presence of Fe(II).

The XRD patterns provide semi-quantitative evidence that Cr(III)-substitution inhibited the Fe(II)-accelerated transformation of schwertmannite. For example, the XRD peak heights for goethite which formed following addition of 1 mM Fe(II) are much lower for the high-Cr(III) system at 7 and 14 days than they are in the corresponding medium-, low- and zero-Cr(III) systems (Fig 5). Likewise, the addition of 10 mM Fe(II) to the medium-, low- and zero-Cr(III) schwertmannite induced substantial transformation of schwertmannite to goethite within just 1 hour, yet the corresponding XRD pattern for the high-Cr(III) system shows negligible goethite formation (Fig 5).

Linear combination fitting of the Fe K-edge EXAFS spectra showed that >75% of solid-phase Fe persisted as schwertmannite under Fe(II)-free conditions (Figs 6 and 7). In comparison, <55% and <30% of solid-phase Fe remained as schwertmannite after 14 days in the presence of 1 and 10 mM Fe(II), respectively (Fig 7 and S2 Table). This provides quantitative verification of the effect of Fe(II) in accelerating schwertmannite transformation under near-neutral conditions.

The Fe K-edge EXAFS results confirm that Cr(III)-substitution inhibited the Fe(II)-accelerated transformation of schwertmannite. For example, following addition of 1 mM Fe(II), only 6% of solid-phase Fe remained as schwertmannite in the zero-Cr(III) system at day 14, compared to 55% in the corresponding high-Cr(III) system (Fig 7). The inhibitory effect of Cr(III)-substitution was lower in the 10 mM Fe(II) treatment, yet schwertmannite transformation in this treatment still decreased from 88% at day 14 in the Cr(III)-free system to 70% in the high-Cr(III) system (Fig 7).

### Changes in Fe(III) and Cr(III) extractability

**Fig 8:**
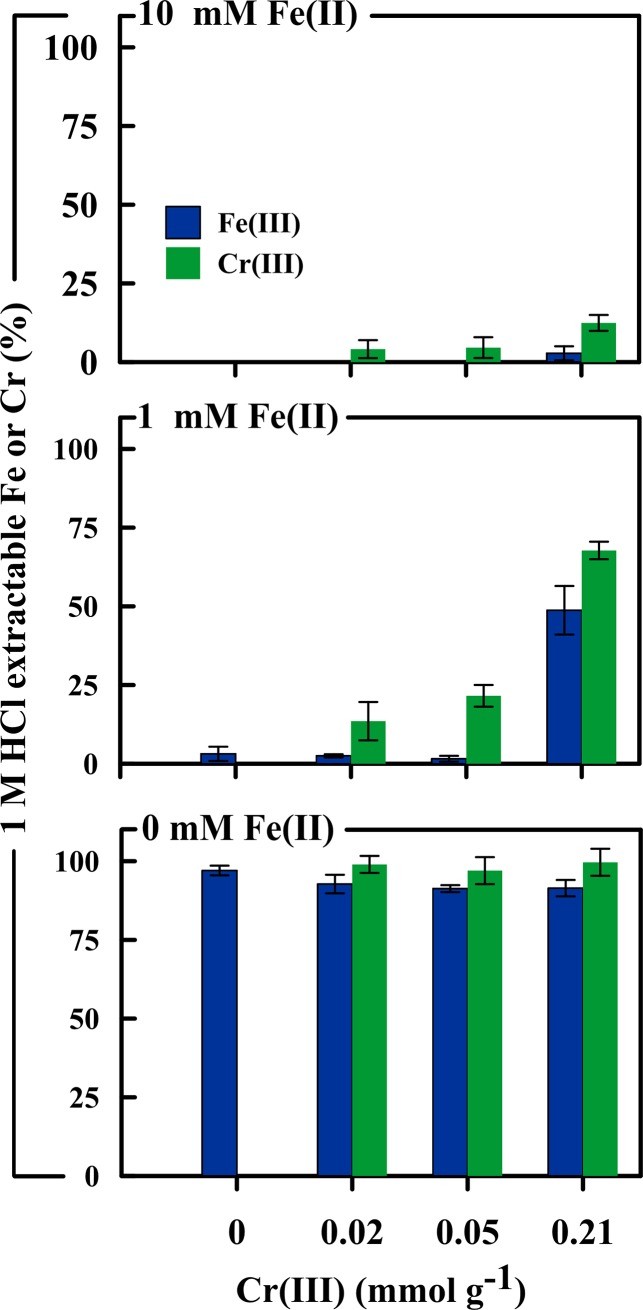
1 M HCl-extractability of Fe and Cr as a function of the level of Cr(III)-substitution after a 14-day reaction period in the presence of 0, 1 or 10 mM Fe(II). Extractability is expressed as a percentage of the total Fe or Cr content.

In the absence of Fe(II), the levels of Fe(III) and Cr(III) extractability in 1 M HCl remained close to 100% and did not vary with the level of Cr(III)-substitution (Fig 8). This is consistent with the day 14 solid-phase in the Fe(II)-free treatments comprising a mixture of mostly schwertmannite and ferrihydrite (Fig 7), both of which are soluble in 1 M HCl.

In treatments which received Fe(II), the extractability of Fe and Cr at day 14 ranged from 0.3–49% and 4–68%, respectively (Fig 8). In general, Fe(III) and Cr(III) extractability varied in accordance with the extent of schwertmannite transformation to highly crystalline goethite, which is only poorly soluble in 1 M HCl. In this case, the lowest levels of extractability were observed in treatments which experienced the largest extent of schwertmannite transformation to goethite (S1 Fig). As such, the HCl-extractability of both Fe and Cr increased with the level of Cr(III)-substitution–due to the effect of Cr(III)-substitution in inhibiting schwertmannite transformation (Fig 8). For example, in the 1 mM Fe(II) treatment, Fe extractability increased from 2.5 to 49% and Cr extractability from 13 to 68% as the amount of substituted Cr(III) increased from 0.02 to 0.21 mmol g^-1^ (Fig 8).

The extractability of Fe(III) versus Cr(III) in the 1 and 10 mM Fe(II) treatments at day 14 was not congruent (Fig 8). More specifically, the extent of Cr(III) extractability was greater than the corresponding extractability of Fe(III). For example, in the 1 mM Fe(II) treatment, the extractability of Cr(III) exceeded that of Fe(III) by between 11 and 19% (Fig 8).

### Mechanism of inhibited schwertmannite transformation

The Fe(II)-accelerated transformation of metastable Fe(III) phases involves the oxidative adsorption of Fe(II) onto the mineral surface. Coupled electron transfer and atom exchange between adsorbed Fe(II) and surface Fe(III) leads to reductive dissolution and the subsequent re-precipitation of Fe(III) as a more stable phase [49–51]. The fact that the Fe(II)-accelerated transformation of schwertmannite involves Fe(III) reductive dissolution implies that the surface concentrations of substituent Cr(III) would have increased wherever Fe(III) dissolution occurred. This is because Cr(III) is insoluble and redox-inactive under the conditions employed in the present study. The theoretical surface enrichment of Cr(III) is consistent with our empirical results showing greater relative extractability of Cr(III) versus Fe(III) in solid-phase samples collected at day 14 (Fig 8). Although not being conclusive proof, the greater extractability of Cr(III) versus Fe(III) supports the hypothesized enrichment of Cr(III) at the surface of the residual schwertmannite.

Any accumulation of Cr(III) at the residual schwertmannite surface will act to physically block Fe sites or divert electron flow. The Fe(II)-induced reductive dissolution of schwertmannite should thus become retarded at a local scale as a result of progressive Cr(III) build-up at the schwertmannite surface. At a bulk scale, this progressive surface enrichment of Cr(III) would therefore produce the observed inhibitory effect of Cr(III)-substitution on schwertmannite transformation.

The present study is the first to show that Cr(III) substitution inhibits the transformation of schwertmannite. However, somewhat comparable findings have been reported in earlier studies on goethite and hematite behaviour. For example, Bousserrhine et al. [52] found that Cr(III)-substitution inhibited the reductive dissolution of goethite and proposed that this may have also been related to Cr(III) accumulation on the goethite surface, which blocked access to sites used in Fe(III) reduction. Analogous results have also been reported by Frierdich et al. [53] who demonstrated that substitution of Cr(III) into goethite and hematite hindered Fe atom exchange between aqueous Fe(II) and structural Fe(III).

### Environmental implications

Naturally occurring schwertmannite is typically enriched in Cr(III), yet the potential effects of Cr(III) substitution on schwertmannite stability have not been previously considered. Here we show, for the first time, that Cr(III) substitution stabilizes schwertmannite against Fe(II)-accelerated transformation to more thermodynamically stable products (predominantly goethite). The Fe(II)-accelerated transformation of schwertmannite is known to strongly influence electron flow in anoxic soils/sediments by altering the favourability of microbial Fe(III) reduction versus SO_4_^2-^ reduction [15]. As such, it is likely that the Cr(III)-substitution may in turn also influence these biogeochemical processes, and their subsequent effects on Fe and S cycling as well as the mobility and fate of associated trace metal(loids). The results also show that schwertmannite transformation to goethite strongly decreases solid-phase Cr(III) extractability, which may translate to a lesser risk of its future oxidation to carcinogenic Cr(VI) if oxidising conditions return.

## Supporting information

S1 Table Chemical composition of the initial schwertmannites. (DOCX)

S2 Table Linear combination fit results for XAS-derived solid-phase Fe speciation (%) during Fe(II) accelerated transformation of Cr(III) incorporated schwertmannite at 14 d. (DOCX)

S1 Fig Relationship between the amount of schwertmannite (%) (quantified through Fe K-edge EXAFS) and 1 M HCl extractable Fe(III) (%) during Fe(II) accelerated transformation at 14 d. (DOCX)
