# Supplementary material for: Chromium(III) substitution inhibits the Fe(II)-accelerated transformation of schwertmannite
Source: PLoS One. 2018 Dec 5;13(12):e0208355. doi: 10.1371/journal.pone.0208355 (PMC6281269; doi:10.1371/journal.pone.0208355)
Supplement: S1 Table — (DOCX) [file pone.0208355.s001.docx]

**S1 Table. Chemical composition of the initial schwertmannites**

|  | **Chromium**  **( mmol g^-1^)** | | **Iron (mmol g^-1^)** | | **Sulfur (mmol g^-1^)** |
| --- | --- | --- | --- | --- | --- |
| Zero Cr(III)-schwertmannite | | 0 | | 8.1 ± 0.42 | 1.63 ± 0.03 |
| Low Cr(III)-schwertmannite | | 0.021± 0.001 | | 8.2 ± 0.08 | 1.70 ± 0.03 |
| Medium Cr(III)-schwertmannite | | 0.05 ± 0.001 | | 8.0 ± 0.24 | 1.71 ± 0.05 |
| High Cr(III)-schwertmannite | | 0.21 ± 0.024 | | 7.9 ± 1.06 | 1.82 ± 0.15 |
