# Supplementary material for: Chromium(III) substitution inhibits the Fe(II)-accelerated transformation of schwertmannite
Source: PLoS One. 2018 Dec 5;13(12):e0208355. doi: 10.1371/journal.pone.0208355 (PMC6281269; doi:10.1371/journal.pone.0208355)
Supplement: S2 Table — (DOCX) [file pone.0208355.s002.docx]

**S2 Table. Linear combination fit results for XAS-derived solid-phase Fe speciation (%) during Fe(II) accelerated transformation of Cr(III) incorporated schwertmannite at 14 d.**

| **Sample name** | **Cr(III) content (mmol g^-1^)** | **Iron minerals (%)** | | | | **R-factor** |
| --- | --- | --- | --- | --- | --- | --- |
|  |  | **Schwertmannite** | **Goethite** | **Lepidocrocite** | **Ferrihydrite** |  |
| **0 Fe(II)** | | | | | | |
| Zero Cr(III)-sch | 0 | 75.5 | 0 | 0 | 24.5 | 0.007 |
| Low Cr(III)-sch | 0.02 | 76.2 | 7.3 | 0 | 16.5 | 0.011 |
| Medium Cr(III)-sch | 0.05 | 89.9 | 0 | 0 | 10.1 | 0.014 |
| High Cr(III)-sch | 0.21 | 100 | 0 | 0 | 0 | 0.021 |
| **1 Fe(II)** | | | | | | |
| Zero Cr(III)-sch | 0 | 5.5 | 70.6 | 23.9 | 0 | 0.003 |
| Low Cr(III)-sch | 0.02 | 24.6 | 75.4 | 0 | 0 | 0.008 |
| Medium Cr(III)-sch | 0.05 | 21.7 | 78.3 | 0 | 0 | 0.023 |
| High Cr(III)-sch | 0.21 | 54.7 | 31.1 | 14.2 | 0 | 0.021 |
| **10 Fe(II)** | | | | | | |
| Zero Cr(III)-sch | 0 | 12 | 88 | 0 | 0 | 0.008 |
| Low Cr(III)-sch | 0.02 | 10 | 90 | 0 | 0 | 0.009 |
| Medium Cr(III)-sch | 0.05 | 13.5 | 86.5 | 0 | 0 | 0.003 |
| High Cr(III)-sch | 0.21 | 29.8 | 70.2 | 0 | 0 | 0.037 |
