# Supplementary material for: Chromium(III) substitution inhibits the Fe(II)-accelerated transformation of schwertmannite
Source: PLoS One. 2018 Dec 5;13(12):e0208355. doi: 10.1371/journal.pone.0208355 (PMC6281269; doi:10.1371/journal.pone.0208355)
Supplement: S1 Fig — (DOCX) [file pone.0208355.s003.docx]

**S1 Fig. Relationship between the amount of schwertmannite (%) (quantified through Fe K-edge EXAFS) and 1 M HCl extractable Fe(III) (%) during Fe(II) accelerated transformation at 14 d.**
